# Systematic Review and Meta-Analysis of Randomized Controlled Trials of Fuke Qianjin Tablet

**DOI:** 10.1155/2021/8861631

**Published:** 2021-02-17

**Authors:** Pan Jie, Zuo Kai-Ni, Wang Xiao-Mei, Zhang Meng-Pei, Wang Zhi-Heng, Zhang Hao-Xiang, Zhu Wen-Tao

**Affiliations:** School of Management, Beijing University of Chinese Medicine, Beijing, China

## Abstract

**Purpose:**

The aim of the research was to evaluate the efficacy and safety associated with Fuke Qianjin tablet combined with conventional therapy in the treatment of pelvic inflammatory diseases and associated complications (endometritis) using a meta-analysis approach. *Patients and Methods*. We searched 8 electronic databases up to December 31, 2019, including PubMed, the Cochrane Library, Embase, Web of Science, CNKI, WanFang, VIP, and SinoMed. Eligible studies were clinical trials of Fuke Qianjin tablet combined with conventional therapy used in the treatment of acute pelvic inflammatory disease, chronic pelvic inflammatory disease, and endometritis. The meta-analysis was performed using STATA15 software.

**Results:**

A total of 125 RCTs (*n* = 14,494) were shortlisted for the meta-analysis, which included 23 trials for acute pelvic inflammatory disease, 69 trials for chronic pelvic inflammatory disease, and 33 trials for endometritis. The overall analysis illustrated Fuke Qianjin tablet combined with conventional therapy was significantly more efficacious than conventional therapy alone across all types of antibiotics treatment for acute pelvic inflammatory disease (OR = 5.57, 95% CI 4.09–7.58, *Z* = 10.90, *p*=0.001), chronic pelvic inflammatory disease (OR = 4.70, 95% CI 4.07–5.42, *Z* = 21.21, *p*=0.001) and endometritis (OR = 5.09, 95% CI 4.03–6.43; *Z* = 13.63, *p*=0.001) in both primary endpoints and secondary endpoints. There is also a trend that Fuke Qianjin tablet combined with conventional therapy has lower adverse reaction rates than conventional therapy alone.

**Conclusion:**

Fuke Qianjin tablet combined with conventional therapy showed better clinical efficacy in the treatment of acute pelvic inflammatory disease, chronic pelvic inflammatory disease, and endometritis. There were no obvious drug-related adverse reactions. Fuke Qianjin tablet presented advantages in shortening the remission time of clinical symptoms, reducing the concentration of serum inflammatory factors, improving endometrial thickness, menstruation, and reducing relapse rate.

## 1. Introduction

Pelvic inflammatory disease (PID), one of the most common infections in women of reproductive age, could lead to endometritis, oophoritis, salpingitis, tuboovarian abscess, peritonitis, and other complications [1–4]. Studies have shown that about 25% of PID patients will have long-term sequelae, including annexitis, irregular menstruation, infertility, ectopic pregnancy, or chronic pelvic pain [5]. Moreover, compared with those without PID, patients with PID had a 1.864 times higher risk of developing preterm labor and a 2.121 times higher risk of developing ectopic pregnancy [6]. Women with PID also have a 6%–8% risk of infertility [5]. Endometritis or complications of PID will further reduce endometrial receptivity and increase the risk of infertility. The prevalence of chronic endometritis in the general population is about 19% [7], and the prevalence of chronic endometritis in infertile patients is about 45% [8]. Studies have shown that effective treatment of endometritis can improve patient reproductive health [9]. A research reported on PID health economics showed that young women are willing to spend 1-2 years of life to prevent PID and its related sequelae [10]. PID has severely affected female reproductive health and has also caused a heavy economic burden. In the United States, the medical cost in treating a PID case is about US$3,200 [11], and the annual PID-related treatment cost on the US healthcare system adds to US$4.2 billion [12]. The current treatment methods for PID are mainly antibiotic treatment and surgical treatment. According to the guidelines [13] issued by CDC in 2015, PID treatment programs must provide empirical and broad-spectrum coverage of possible pathogens. However, long-term, repeated use of antibiotics may lead to increasing antibiotic resistance of related pathogens [14]. According to the 2018 World Health Organization (WHO) report, pathogens showed high rates of quinolone resistance, increasing azithromycin resistance, and emerging resistance to extended-spectrum cephalosporins [15]. On the other end, surgical treatment is only carried out when drug treatment is ineffective or emergency symptoms appear. Surgery presents risk of intrauterine iatrogenic damage, and so, surgical treatment measures should be carefully considered specific to the conditions of patients [16].

Fuke Qianjin tablet is a pure Chinese medicine preparation approved by the National Medical Products Administration. It is widely used clinically to treat acute pelvic inflammatory disease, chronic pelvic inflammatory disease, and endometritis. In recent years, many researchers have performed evaluation of the clinical application of Fuke Qianjin tablets [17], but further evaluation is deemed necessary. Therefore, this study aims to comprehensively evaluate the efficacy and safety of Fuke Qianjin tablets and provide a basis for its application as a treatment for pelvic inflammatory disease.

## 2. Materials and Methods

### 2.1. Literature Search

This study evaluated the clinical effectiveness and safety of Fuke Qianjin tablets in the treatment of acute pelvic inflammatory disease, chronic pelvic inflammatory disease, and endometritis. Synonyms of Fuke Qianjin tablets such as “Fuke Qianjin tablet,” “Qianjin tablet,” and “Qianjin” were used to search English databases. Chinese database search strategies and results are shown in Table 1. Additional, a manual search on chictr.org.cn (clinical trial database in China) was also done to identify potential unpublished studies.

### 2.2. Inclusion and Exclusion Criteria

Eligible studies should have met the following criteria: (1) randomized controlled trials (RCTs) of Fuke Qianjin tablets published in China or internationally; (2) patients diagnosed with acute pelvic inflammatory disease, chronic pelvic inflammatory disease, or endometritis; (3) the intervention measures of the experimental group are Fuke Qianjin tablets alone or combined with conventional treatment; and (4) the study reported one or more of the following outcome indicators: the main outcome indicators such as total effective rate and incidence of adverse reactions, and secondary outcome indicators such as clinical symptom relief time, endometrial thickness, serum inflammatory factor concentration, menstrual recovery, and relapse rate.

Exclusion criteria were (1) nonpharmacological treatment-related studies, such as acupuncture, physical therapy, and hot compresses; (2) studies with repeated publications; (3) studies with completely missing data; and (4) studies with unclear interventions, unclear description of efficacy evaluation criteria, or statistical errors.

### 2.3. Quality Assessment

Two authors independently evaluated the risk of bias of the included literature using the Cochrane Handbook which consists of seven domains [18]: random sequence generation, allocation concealment, blinding of participants and personnel, blinding of outcome assessments, incomplete outcome data, selective reporting, and other bias. For each domain, the risk of bias for each study was assessed according to three categories: low risk, high risk, or unclear risk. Disagreements encountered during the process were settled by a third author through discussion.

### 2.4. Data Extraction

Two independent reviewers extracted the following data from the included studies using a standardized data extraction form. Data are connected with study characteristics (first author's name, the sample size of each group, and publication year), patient characteristics (age and course of the disease), treatment measures, outcome measures, and literature quality evaluation information. Any disagreement between the two reviewers was subject to a discussion or further inquiry from the third researcher.

### 2.5. Statistical Analysis

All analyses were performed using STATA version 15.0. For dichotomous data, we calculated odds ratio (OR) with 95% CI; for continuous outcome data, we calculated the standard mean difference (SMD) with 95% CI. In this case, the Cochrane *Q* statistic and *I*^*2*^ statistics were conducted to test the heterogeneity across the included studies [19–22], in which *p* value <0.1 or *I*^*2*^ >50% was deemed to represent substantial heterogeneity; then, the data were pooled using a random-effect model. On the contrary, the fixed-effect model is adopted. Publication bias was assessed by directly observing the symmetry of the inverted funnel chart, using the Harbord test and Egger's test [23–25]. In the case of significant publication bias, the trim and fill method [26] was used to correct the effect to verify the robustness of the meta-analysis results.

## 3. Results

### 3.1. Retrieval Results and Quality Assessment

**Figure 1:**
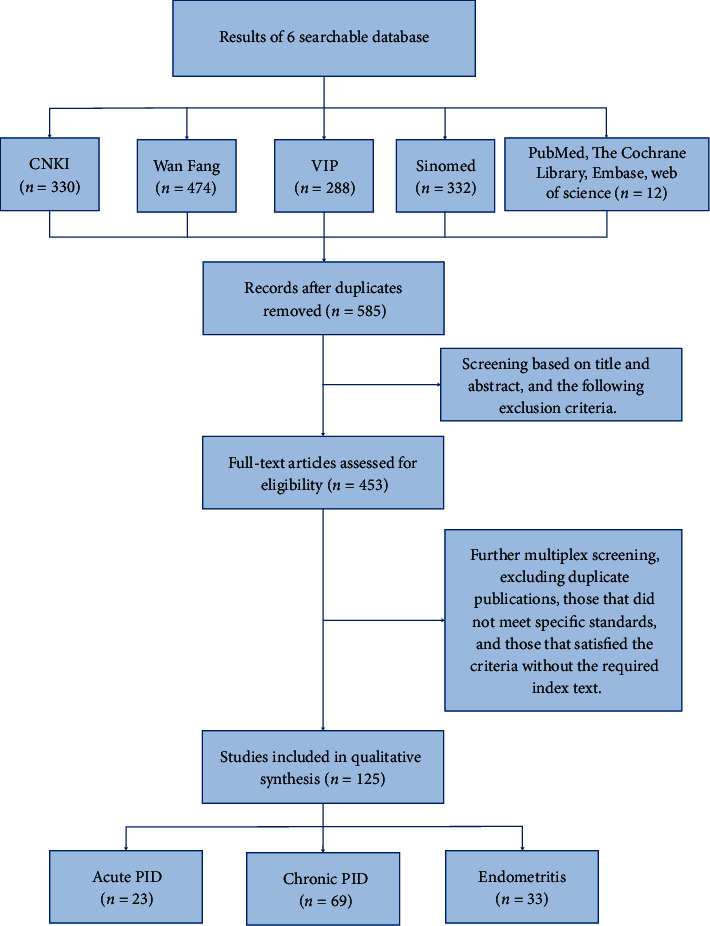
Study selection steps.

All the retrieved literature (*n* = 1,434) were imported into the NoteExpress V3.2.0 version (NoteExpress, Beijing Aegean Sea Lezhi Technology Co., Ltd.). A 95% accuracy algorithm was set to assess duplicates, and then, additional duplicate checks were performed manually. After screening with inclusion and exclusion criteria, a total of 125 separate studies were included for the final analysis (Figure 1), including 23 for acute pelvic inflammatory disease [27–49], 69 for chronic pelvic inflammatory disease [50–118], and 33 for endometritis [119–151]. The full list of studies is illustrated in Tables 1–3.

### 3.2. Meta-Analysis of Fuke Qianjin Tablet in the Treatment of Acute Pelvic Inflammatory

#### 3.2.1. The Main Outcome

**Figure 2:**
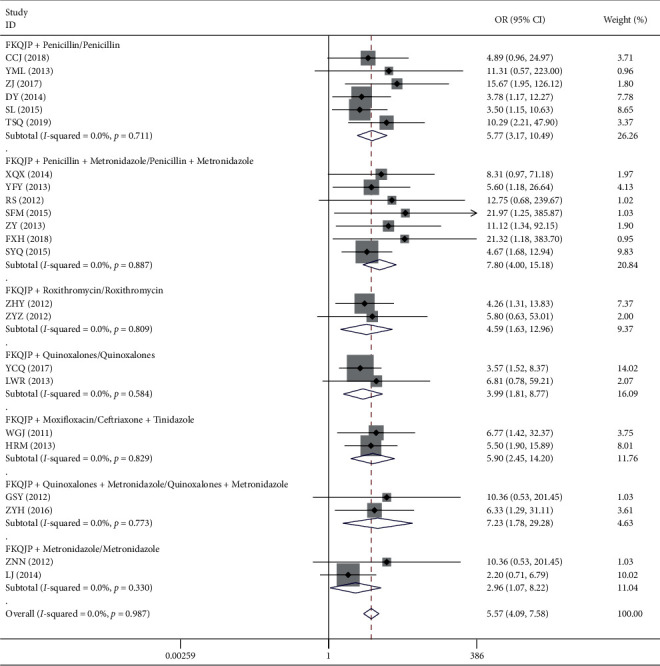
Meta-analysis of total effective rate of acute pelvic inflammatory disease.

*(1). Total of Effective Rate*. There were 23 articles [27–49] about Fuke Qianjin tablets combined with antibiotics in the treatment of acute pelvic inflammatory disease. The results showed that Fuke Qianjin tablets combined with antibiotics led to significantly better clinical efficacy compared to antibiotics alone (OR = 5.57, 95% CI 4.09–7.58, *Z* = 10.90, *p*=0.001). The results of the subgroup analysis showed that the efficacy of FKQJP combined with antibiotics was not affected by antibiotic types (Table 4 and Figure 2).

**Figure 3:**
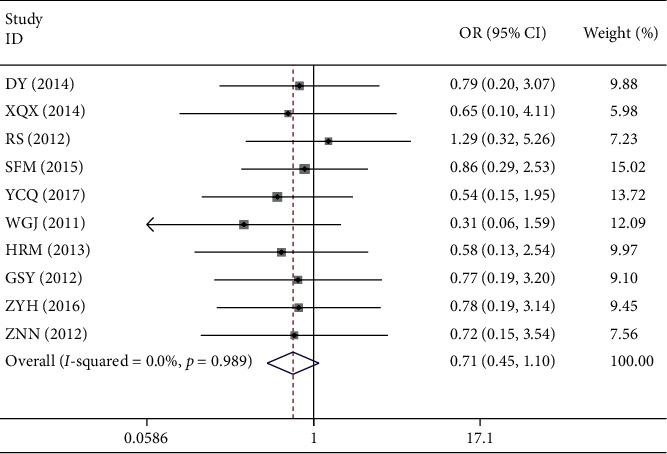
Meta-analysis of adverse reaction of acute pelvic inflammatory disease.

*(2). Adverse Reaction*. A total of 15 studies reported outcome of adverse reactions, in which 10 studies reported the specific data of 90 cases of adverse reactions. The difference of the incidence of the adverse reaction between the two groups was not statistically significant (OR = 0.71, 95% CI 0.45–1.10, *Z* = 1.55, *p*=0.121). 5 studies reported no adverse events or mild adverse reactions which improved spontaneously (Table 4 and Figure 3).

#### 3.2.2. Secondary Outcomes

Comparisons in secondary outcomes were also made. Similar to the primary outcome, Fuke Qianjin tablets combined with antibiotics had better efficacy profiles in shortening the remission time of clinical symptoms and reducing serum inflammatory factor concentrations when compared with antibiotics alone, as shown in Table 5.

#### 3.2.3. Publication Bias

**Figure 4:**
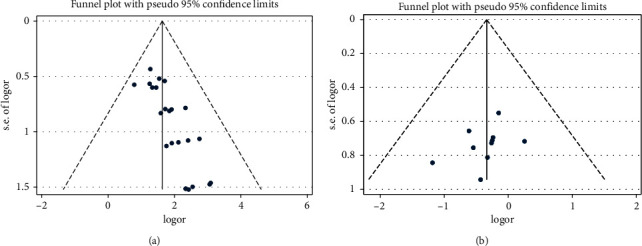
(a) Funnel plot of acute pelvic inflammatory disease of total effective rate. (b) Funnel plot of acute pelvic inflammatory disease of incidence of adverse reaction.

The publication bias test was performed on the total effective rate and incidence of adverse reactions, and the inverted funnel diagram (Figures 4(a) and 4(b)) was drawn, respectively. The results of the Harbord test showed that there were significant publication bias and small sample effect in the outcome of the total effective rate (*t* = 5.95, *p*=0.001), and there were not significant publication bias and small sample effect in the outcome of the incidence of adverse reactions (*t* = −0.57, *p*=0.583).

The sensitivity analysis was carried out by using the trim and filled method. The point estimated value and 95% confidence interval of the summary effect OR before and after the implementation of the trim and fill method was 5.57[4.09, 7.58] and 4.25 [3.18, 5.68], respectively. The differences between two of groups were still statistically significant (*Z* = 9.765, *p*=0.001). The iterative results show that 10 additional studies are needed to further enhance the stability of the research results.

### 3.3. Meta-Analysis of Fuke Qianjin Tablet in the Treatment of Chronic Pelvic Inflammatory

#### 3.3.1. The Main Outcome

**Figure 5:**
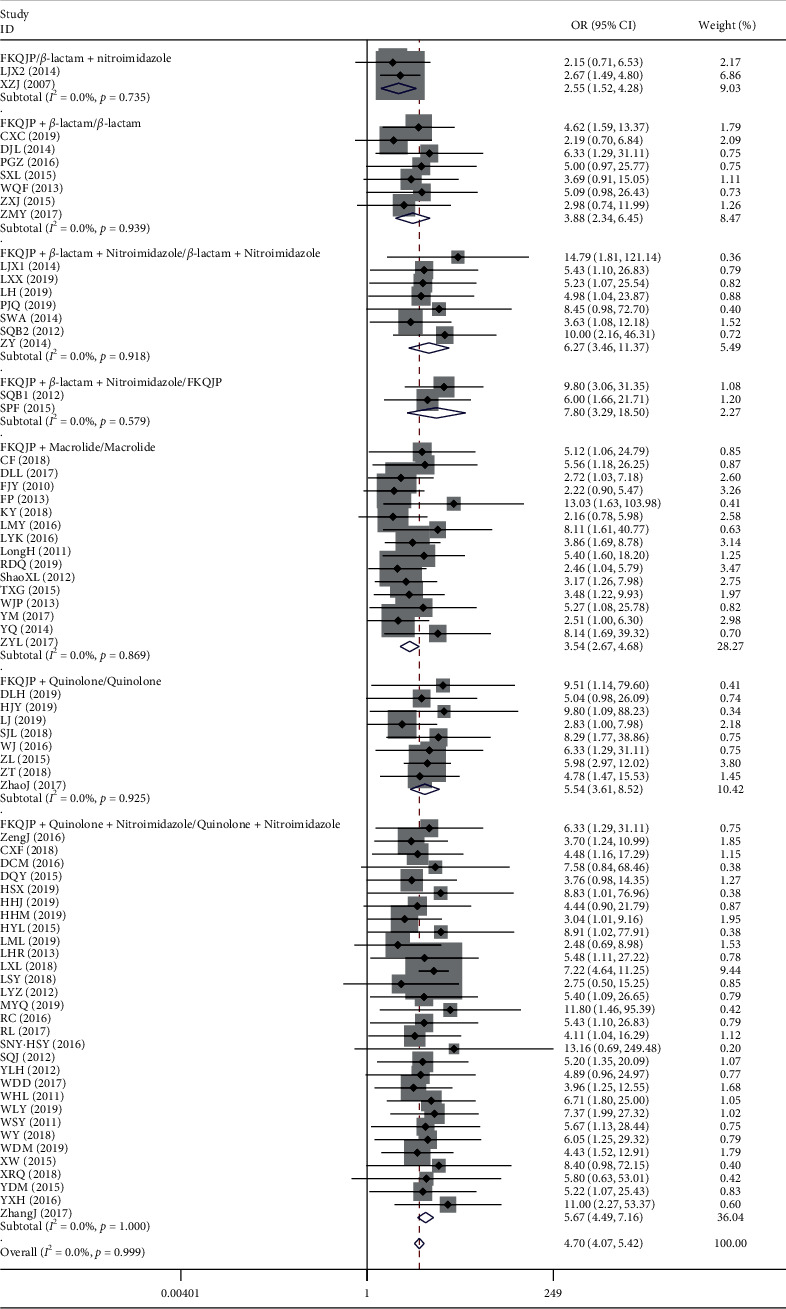
Meta-analysis of total effective rate of chronic pelvic inflammatory disease.

*(1). Total of Effective Rate*. There were 69 articles about Fuke Qianjin tablets combined with antibiotics in the treatment of chronic pelvic inflammatory disease [50–118]. The results showed that Fuke Qianjin tablets combined with antibiotics had better clinical efficacy (OR = 4.70, 95% CI 4.07–5.42, *Z* = 21.21, *p*=0.001) than antibiotics alone. The results of the subgroup analysis showed that the efficacy of Fuke Qianjin tablets combined with antibiotics was not affected by antibiotic types (Table 4 and Figure 5).

**Figure 6:**
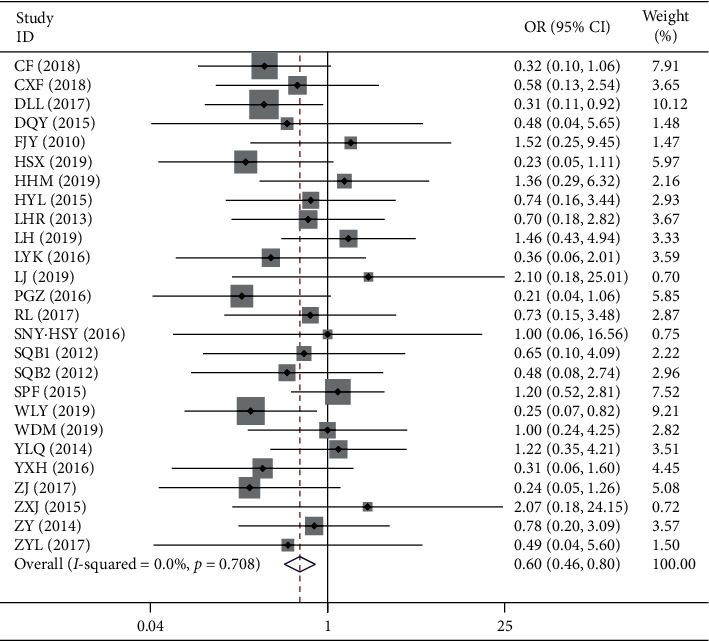
Meta-analysis of adverse reaction of chronic pelvic inflammatory disease.

*(2). Adverse Reaction*. A total of 30 studies reported adverse reactions to the treatment of chronic pelvic inflammation. Among them, 26 studies reported specific data. The fixed-effects model analysis showed that compared with the control group, the incidence of adverse reactions in the combined group of Fuke Qianjin tablets was lower (OR = 0.60, 95% CI 0.46–0.80, *Z* = 3.55, *p*=0.001). (Table 4 and Figure 6).

#### 3.3.2. Secondary Outcomes

Comparisons in secondary outcomes were also made. Similar to the primary outcome, Fuke Qianjin tablets combined with antibiotics had better efficacy profiles in shortening the remission time of clinical symptoms and reducing serum inflammatory factor concentrations when compared with antibiotics alone, as shown in Table 5.

#### 3.3.3. Publication Bias

**Figure 7:**
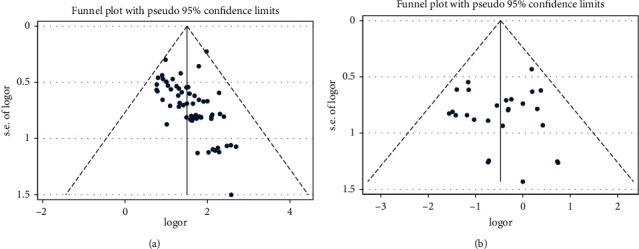
(a) Funnel plot of chronic pelvic inflammatory disease of the total effective rate. (b) Funnel plot of chronic pelvic inflammatory disease of incidence of adverse reaction.

Perform the publication bias test on total effective rate and the incidence of adverse reactions, and the inverted funnel diagram (Figures 7(a) and 7(b)) was drawn, respectively. The inverted funnel plots of the 2 outcomes were substantially symmetric. The results of the Harbord test showed that there were no significant publication bias and small sample effect in the total effective rate (*t* = 0.11, *p*=0.912) and adverse reaction rate (*t* = 0.96, *p*=0.349).

### 3.4. Meta-Analysis of Fuke Qianjin Tablet in the Treatment of Endometritis

#### 3.4.1. The Main Outcome

**Figure 8:**
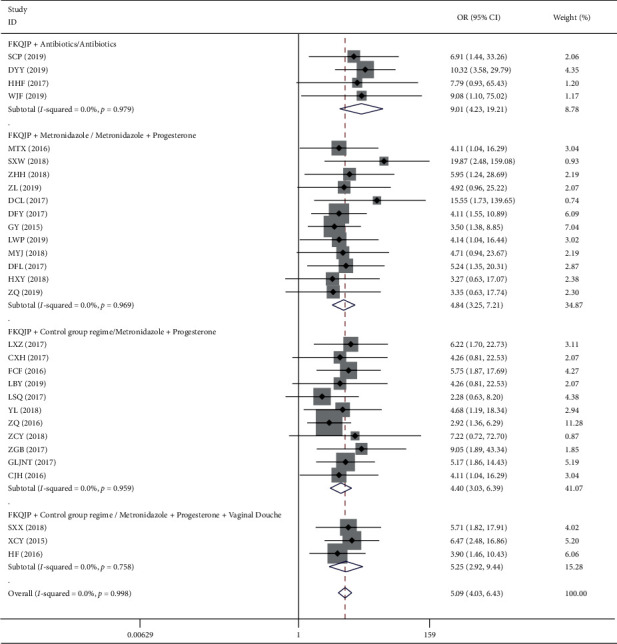
Meta-analysis of total effective rate of endometritis disease.

*(1). Total of Effective Rate*. There were 33 studies about Fuke Qianjin tablets combined with conventional treatment of endometritis, and 30 studies reported the total effective outcome. The results of the fixed-effect model showed that the combined group of Fuke Qianjin tablets had a better total effective rate of treating endometritis than the control group (OR = 5.09, 95% CI 4.03–6.43, *Z* = 13.63, *p*=0.001). According to the specific intervention measures, the subgroup analysis showed that the combined group of Fuke Qianjin tablets had a better curative effect than the control group (Table 4 and Figure 8).

*(2). Adverse Reaction*. In the included study, only 4 studies (a total of 427 participants) counted adverse reactions as part of the study. 3 studies reported no serious adverse reactions or no obvious adverse reactions, and 1 reported specific adverse reactions: there were 2 cases in the trial group (1 case of nausea and 1 case of gastrointestinal reaction); there were 3 cases in the control group (1 case of nausea, 1 case of vomiting, and 1 case of gastrointestinal reaction). There was no significant difference in the incidence of adverse reactions between the two groups (*p* > 0.05).

#### 3.4.2. Secondary Outcomes

Comparisons in secondary outcomes were also made. Similar to the primary outcome, Fuke Qianjin tablets combined with antibiotics had better efficacy profiles in improving endometrial thickness, menstrual recovery, and relapse rate, as shown in Table 5.

#### 3.4.3. Publication Bias

**Figure 9:**
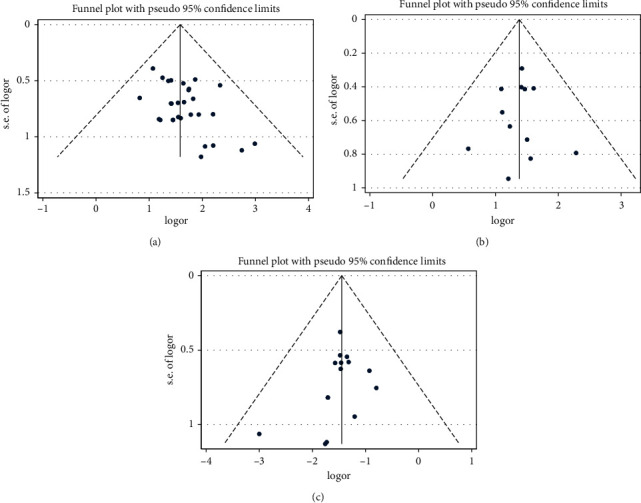
(a) Funnel plot of endometritis of the total effective rate. (b) Funnel plot of endometritis of menstruation recovery rate. (c) Funnel plot of endometritis of incidence of irregular vaginal bleeding.

Perform the publication bias test on total effective rate, endometrial thickness, menstrual normalization rate, and the incidence of irregular vaginal bleeding, and the inverted funnel diagram (Figures 9(a)–9(c)) was drawn, respectively. The results of the Harbord test showed that there were no significant publication bias and small sample effect in the total effective rate (*t* = 0.17, *p*=0.859), endometrial thickness (*t* = 2.04, *p*=0.060), menstrual normalization (*t* = −0.90, *p*=0.39), and the incidence of irregular vaginal bleeding (*t* = 0.17, *p*=0.867). The inverted funnel plots of the 3 outcomes were substantially symmetric.

## 4. Discussion

In recent years, some researchers have evaluated Fuke Qianjin tablets in the treatment of acute pelvic inflammation, chronic pelvic inflammation, and endometritis [17,152–154]. It is considered that the clinical effect of Fuke Qianjin tablets combined with antibiotics is better than that of antibiotics alone, which is consistent with the conclusion of this study. The purpose of this study was to compare the efficacy and safety of gynecological Qianjin tablets combined with routine treatment in patients with pelvic inflammatory diseases. The results of this study showed that Fuke Qianjin tablets combined with conventional treatment have better efficacy outcomes for acute pelvic inflammatory disease, chronic pelvic inflammatory disease, and endometritis when compared to using antibiotics alone. Moreover, it can shorten the time of clinical symptom relief, reduce serum inflammatory factor concentration, improve endometrial thickness, improve menstruation, and reduce relapse rate. According to studies reports, the combined application of Fuke Qianjin tablets has no significant adverse reactions, suggesting a strong safety profile.

The occurrence of pelvic inflammatory disease is not only related to a single bacterium. Studies have shown that 30–40% of pelvic inflammatory cases are caused by multibacterial infection [155]. Therefore, it is necessary to provide broad-spectrum coverage of possible pathogens during treatment. The results of the subgroup analysis showed that the efficacy of Fuke Qianjin tablets combined with antibiotics was not affected by antibiotic types. It demonstrated that Fuke Qianjin tablets are effective in the treatment of a variety of bacteria and have the therapeutic effect of broad-spectrum coverage. Evidence from the PEACH research suggests that short-term and long-term outcomes of oral and intravenous regimens are similar [156], so there is no subgroup analysis of antibiotic administration routes and courses in this study. In addition, two studies of Fuke Qianjin tablets compared with antibiotics (penicillin + metronidazole) showed that Fuke Qianjin tablet was more effective in the treatment of chronic pelvic inflammation. Due to the small number of literature included, more clinical studies need to be carried out to verify the clinical efficacy of Fuke Qianjin tablets alone.

Traditional Chinese medicine has multiple components, and its complexity means it has many potential targets. This makes it more difficult for bacteria to become resistant to it, which has a definite clinical advantage for PID. Fuke Qianjin tablet is extracted from herbs including *Suberect spatholobus* stem, Jin Ying Gen, *Angelica sinensis*, and *Andrographis paniculata* Nees. *Suberect spatholobus* stem has the effect of relaxing muscles and activating blood circulation; Jin Ying Gen has the effect of fixing essence and astringent intestines; *Angelica sinensis* has the effect of tonifying blood, harmonizing blood, regulating menstruation, and stopping bleeding; *Andrographis paniculata* Nees has the effect of cooling blood and detumescence, clearing heat, and detoxification. Related animal experiments and modern pharmacological studies [157–161] showed that Fuke Qianjin tablets may achieve the anti-inflammatory effect by regulating the concentration of serum TNF-*α*, NF-kB, IL-2, IL-6, and other factors to reduce the inflammatory response mediated by cellular inflammatory factors. It can also promote the production of IgA, IgG, and IgM [162], improve the body's immunity, play antibacterial and anti-inflammatory effects, and also improve the body's ability to resist infection. This demonstrated that in the future, due to the increasing resistance of pathogens to antibiotics, traditional Chinese medicine may play a more important role in the treatment of PID anti-inflammatory. Therefore, it is necessary to carry out a clinical study on the graded dose response of Fuke Qianjin tablets combined with antibiotics to explore whether the combined application can reduce the dose and frequency of antibiotics, so as to alleviate the situation of reduced efficacy due to increased antibiotic resistance.

Heterogeneity between studies is a key issue in meta-analysis. The existence of heterogeneity has an impact on the merger of research results and also directly affects the interpretation of meta-analysis results. This research conducted a subgroup analysis and sensitivity analysis on the outcomes with high heterogeneity. Through a reexamination of studies with high heterogeneity and comparison with other studies, the stability of the sensitive analysis results to determine the credibility of the research results and whether to retain the studies. In the meta-analysis of acute pelvic inflammatory disease, there was significant heterogeneity in some secondary outcome indicators, which was mainly caused by Zhao and Huang [29] and Fan [38]. The reverification of the two articles showed that the literature quality evaluation was moderately biased risk. After excluding the literature data, the result of meta-analysis was still significant, and the sensitivity analysis showed that the result was stable. In the meta-analysis of chronic pelvic inflammation, two secondary outcome indicators of the remission time of uterine pain and remission time of abdominal pain carried out subgroup analysis according to the course of treatment. The results showed that the heterogeneity significantly decreased, and the difference between the experimental group and the control group was still statistically significant. It can be considered that the results of meta-analysis of these two indicators were robust. Sensitivity analysis was performed on the two secondary outcome indicators of CRP and TNF-*α* concentration. The outcome indicator of TNF-*α* concentration has passed the sensitivity analysis, and the meta-analysis results of this indicator can be considered robust. The outcome indicator of CRP concentration in chronic pelvic inflammatory disease cannot pass the sensitivity analysis. It is considered that the reliability of the result of meta-analysis of this indicator was low, and the source of its heterogeneity needs to be further discussed.

This research still has certain limitations. First of all, affected by the quality of the original literature, only 2 articles of the original literature reported the specific implementation of the blind method, and most of them did not report the specific implementation of the random method and the blind method, which may have some potential bias. Second, the subgroup analysis and sensitivity analysis of the secondary outcome indicators with heterogeneity showed that the results were robust. But the number of secondary outcome indicators reported in articles was very small, thereby potentially affecting the strength of the results. Finally, the clinical research of gynecological Fuke Qianjin tablets is only carried out across hospitals in China. The extrapolation of the results on an international scale maybe limited. In order to overcome the above limitations and verify the results of this study, additional high-quality randomized controlled trials that employ a larger sample size are required.

## 5. Conclusion

In order to systematically evaluate the efficacy of Fuke Qianjin tablets in the treatment of pelvic inflammatory diseases and endometritis, this study uses indicators such as total effective rate and incidence of adverse reactions to compare with the combined application of various conventional treatment programs. Results from 125 studies have been observed in the treatment of acute pelvic inflammatory disease, chronic pelvic inflammatory disease, and endometritis. Overall, these studies report positive effects of Fuke Qianjin tablets as adjuvant medication. In addition, the incidence of adverse reactions was not statistically different in the analyzed studies.

## Figures and Tables

**Table 1:** Chinese database retrieval strategy and results.

Database	Search strategy	Outcome
CNKI	(SU=('Pelvic inflammatory disease'+'Chronic pelvic inflammatory disease'+' Pelvic inflammatory sequelae '+”)∗('Fuke Qianjin Tablet ')) NOT TI=('rat')	252
WanFang	((subject:(“Pelvic inflammatory disease”+“Chronic pelvic inflammatory disease”+“Pelvic inflammatory sequelae”+“acute pelvic inflammatory disease”)∗ (“Fuke Qianjin Tablet”)) not subject：(“rat”+“rabbit”+“Meta”))∗Date:-2019	387
VIP	(M=(“Pelvic inflammatory disease”+“Chronic pelvic inflammatory disease”+“Pelvic inflammatory sequelae”+“acute pelvic inflammatory disease”) AND (acute pelvic inflammatory disease “Fuke Qianjin Tablet”)	214
SinoMed	(“Fuke Qianjin Tablet” Djangoorm: Intelligence AND(“Pelvic inflammatory disease” Djangoorm: Intelligence OR “Chronic pelvic inflammatory disease” Djangoorm: Intelligence OR “Pelvic inflammatory sequelae” Djangoorm: Intelligence OR “acute pelvic inflammatory disease” Djangoorm: Intelligence)) NOT(“rat” Djangoorm: Intelligence)	278

CNKI	(SU=('endometritis'+'chronic endometritis'+'Acute endometritis'+'puerperal endometritis'+'puerperium endometritis')∗(' Fuke Qianjin Tablet ')) NOT TI=('rat')	78
WanFang	((subject: (“endometritis”+“chronic endometritis”+“Acute endometritis”+“puerperal endometritis”+“puerperium endometritis”)∗ (“Fuke Qianjin Tablet”)) not subject：(“rat”+“rabbit”+“Meta”))∗Date:-2019	87
VIP	(M=(“endometritis”+“chronic endometritis”+“Acute endometritis”) AND (“Fuke Qianjin Tablet”)	74
SinoMed	(“Fuke Qianjin Tablet” Djangoorm: Intelligence AND (“endometritis” Djangoorm: Intelligence OR “chronic endometritis” Djangoorm: Intelligence OR “Acute endometritis” Djangoorm: Intelligence OR “puerperal endometritis” Djangoorm: Intelligence OR “puerperium endometritis” Djangoorm: Intelligence)) NOT(“rat” Djangoorm: Intelligence OR “meta” Djangoorm: Intelligence)	54

**Table 2:** Characteristics of the studies included in this meta-analysis.

Author	Na/Nb	Course	Experimental	Control	Outcome
*Acute pelvic inflammatory*					
CCJ (2018) [27]	35/35	15	FKQJP + C	Penicillin	①③
YML (2013) [28]	24/23	12	FKQJP + C	Penicillin	①
ZJ (2017) [29]	48/48	15	FKQJP + C	Penicillin	①③
DY (2014) [30]	68/68	10∼15	FKQJP + C	Penicillin	①②③④
SL (2015) [31]	50/50	10	FKQJP + C	Penicillin	①②④
TSQ (2019) [32]	50/50	10	FKQJP + C	Penicillin	①
XQX (2014) [33]	39/39	15	FKQJP + C	Penicillin + metronidazole	①②③
YFY (2013) [34]	67/68	10∼15	FKQJP + C	Penicillin + metronidazole	①③
RS (2012) [35]	36/36	10	FKQJP + C	Penicillin + metronidazole	①②
SFM (2015) [36]	66/66	10	FKQJP + C	Penicillin + metronidazole	①②
ZY (2013) [37]	43/43	10	FKQJP + C	Penicillin + metronidazole	①④
FXH (2018) [38]	39/39	14	FKQJP + C	Penicillin + metronidazole	①②③④
SYQ (2015) [39]	110/110	15	FKQJP + C	Penicillin + metronidazole	①③
ZHY (2012) [40]	60/60	10	FKQJP + C	Roxithromycin	①②
ZYZ (2012) [41]	30/30	12	FKQJP + C	Roxithromycin	①②
YCQ (2017) [42]	60/60	7∼14	FKQJP + C	Moxifloxacin	①②
LWR (2013) [43]	43/43	10	FKQJP + C	Levofloxacin hydrochloride	①③④
WGJ (2011) [44]	50/50	14	FKQJP + C	Ceftriaxone + tinidazole	①②
HRM (2013) [45]	60/60	7∼15	FKQJP + C	Ceftriaxone + tinidazole	①②
GSY (2012) [46]	30/30	10	FKQJP + C	Levofloxacin lactate + metronidazole	①②
ZYH (2016) [47]	40/40	7∼14	FKQJP + C	Gatifloxacin + metronidazole	①②
ZNN (2012) [48]	30/30	12	FKQJP + C	Metronidazole	①②
LJ (2014) [49]	36/36	14	FKQJP + C	Metronidazole	①④

*Chronic pelvic inflammatory*					
LJX2 (2014) [50]	40/40	42	FKQJP	*β*-Lactam + nitroimidazole	①
XZJ (2007) [51]	102/102	45/21	FKQJP	*β*-Lactam + nitroimidazole	①
CXC (2019) [52]	64/64	21	FKQJP + C	*β*-Lactam	①④
DJL (2014) [53]	62/62	21	FKQJP + C	*β*-Lactam	①②
PGZ (2016) [54]	40/40	14	FKQJP + C	*β* -Lactam	①②③
SXL (2015) [55]	32/32	21	FKQJP + C	*β*-Lactam	①③
WQF (2013) [56]	35/35	20	FKQJP + C	*β*-Lactam	①
ZXJ (2015) [57]	30/30	14	FKQJP + C	*β*-Lactam	①②
ZMY (2017) [58]	50/50	14	FKQJP + C	*β*-Lactam	①②
LJX1 (2014) [50]	40/40	42	FKQJP + C	*β*-Lactam + nitroimidazole	①
LXX (2019) [59]	43/43	15	FKQJP + C	*β*-Lactam + nitroimidazole	①
LH (2019) [60]	52/52	14	FKQJP + C	*β*-Lactam + nitroimidazole	①②
PJQ (2019) [61]	75/75	15	FKQJP + C	*β*-Lactam + nitroimidazole	①
SWA (2014) [62]	36/36	28	FKQJP + C	*β*-Lactam + nitroimidazole	①
SQB2 (2012) [63]	50/50	28	FKQJP + C	*β*-Lactam + nitroimidazole	①②
ZY (2014) [64]	54/54	28	FKQJP + C	*β*-Lactam + nitroimidazole	①②
SQB1 (2012) [63]	50/50	28	FKQJP+*β*-lactam + nitroimidazole	FKQJP	①②
SPF (2015) [65]	75/75	14	FKQJP+*β*-lactam + nitroimidazole	FKQJP	①②
CF (2018) [66]	60/60	42	FKQJP + C	Macrolide	①②
DLL (2017) [67]	81/81	42	FKQJP + C	Macrolide	①②
FJY (2010) [68]	63/63	15	FKQJP + C	Macrolide	①②
FP (2013) [69]	62/62	14	FKQJP + C	Macrolide	①
KY (2018) [70]	100/86	14	FKQJP + C	Macrolide	①③
LMY (2016) [71]	50/50	14	FKQJP + C	Macrolide	①
LYK (2016) [72]	30/30	15	FKQJP + C	Macrolide	①②
LongH (2011) [73]	90/90	28	FKQJP + C	Macrolide	①
RDQ (2019) [74]	40/40	21	FKQJP + C	Macrolide	①
ShaoXL (2012) [75]	87/86	15	FKQJP + C	Macrolide	①
TXG (2015) [76]	90/90	15	FKQJP + C	Macrolide	①②
WJP (2013) [77]	52/48	51	FKQJP + C	Macrolide	①
YM (2017) [78]	50/50	51	FKQJP + C	Macrolide	①
YQ (2014) [79]	67/67	15	FKQJP + C	Macrolide	①②
ZYL (2017) [80]	40/40	20	FKQJP + C	Macrolide	①②
DLH (2019) [81]	45/45	30	FKQJP + C	Quinolone	①
HJY (2019) [82]	31/31	30	FKQJP + C	Quinolone	①④
LJ (2019) [83]	22/22	14	FKQJP + C	Quinolone	①②
SJL (2016) [84]	53/53	30	FKQJP + C	Quinolone	①
WJ (2018) [85]	40/40	14	FKQJP + C	Quinolone	①
ZL (2015) [86]	40/40	21	FKQJP + C	Quinolone	①
ZT (2018) [87]	160/160	21	FKQJP + C	Quinolone	①④
ZhaoJ (2017) [88]	55/55	28	FKQJP + C	Quinolone	①④
ZengJ (2016) [89]	40/40	14	FKQJP + C	Quinolone + nitroimidazole	①②
CXF (2018) [90]	58/58	42	FKQJP + C	Quinolone + nitroimidazole	①③
DCM (2016) [91]	47/47	28	FKQJP + C	Quinolone + nitroimidazole	①③
DQY (2015) [92]	25/25	28	FKQJP + C	Quinolone + nitroimidazole	①②
HSX (2019) [93]	65/65	42	FKQJP + C	Quinolone + nitroimidazole	①②
HHJ (2019) [94]	60/60	28	FKQJP + C	Quinolone + nitroimidazole	①
HHM (2019) [95]	63/63	28	FKQJP + C	Quinolone + nitroimidazole	①②③④
HYL (2015) [96]	60/60	32	FKQJP + C	Quinolone + nitroimidazole	①②
LML (2019) [97]	29/29	14	FKQJP + C	Quinolone + nitroimidazole	①③
LHR (2013) [98]	45/41	28	FKQJP + C	Quinolone + nitroimidazole	①②③
LXL (2018) [99]	41/41	14	FKQJP + C	Quinolone + nitroimidazole	①④
LSY (2018) [100]	587/588	28	FKQJP + C	Quinolone + nitroimidazole	①③④
LYZ (2012) [101]	35/35	28	FKQJP + C	Quinolone + nitroimidazole	①③
MYQ (2019) [102]	30/30	28	FKQJP + C	Quinolone + nitroimidazole	①
RC (2016) [103]	60/60	14	FKQJP + C	Quinolone + nitroimidazole	①
RL (2017) [104]	43/43	28	FKQJP + C	Quinolone + nitroimidazole	①②
SNY·HSY (2016) [105]	40/40	20	FKQJP + C	Quinolone + nitroimidazole	①②
SQJ (2012) [106]	30/30	14	FKQJP + C	Quinolone + nitroimidazole	①②
YLH (2012) [107]	42/42	28	FKQJP + C	Quinolone + nitroimidazole	①③④
WDD (2017) [108]	35/35	28	FKQJP + C	Quinolone + nitroimidazole	①
WHL (2011) [109]	90/90	28	FKQJP + C	Quinolone + nitroimidazole	①③
WLY (2019) [110]	50/50	30	FKQJP + C	Quinolone + nitroimidazole	①②
WSY (2011) [111]	50/50	90	FKQJP + C	Quinolone + nitroimidazole	①
WY (2018) [112]	36/36	28∼42	FKQJP + C	Quinolone + nitroimidazole	①
WDM (2019) [113]	48/48	28	FKQJP + C	Quinolone + nitroimidazole	①②
XW (2015) [114]	65/63	28	FKQJP + C	Quinolone + nitroimidazole	①③
XRQ (2018) [115]	37/37	28	FKQJP + C	Quinolone + nitroimidazole	①③
YDM (2015) [116]	30/30	28	FKQJP + C	Quinolone + nitroimidazole	①②
YXH (2016) [117]	53/53	42	FKQJP + C	Quinolone + nitroimidazole	①②
ZhangJ (2017) [118]	35/35	14	FKQJP + C	Quinolone + nitroimidazole	①②

*Endometritis*					
SFF (2017) [119]	44/44	45	FKQJP + C	Cefoxitin + doxycycline	④
SCP (2019) [120]	46/46	45	FKQJP + C	Cefoxitin + doxycycline	①
DYY (2019) [121]	174/174	45	FKQJP + C	Cefoxitin + doxycycline	①③④
LY (2019) [122]	34/33	45	FKQJP + C	Cefoxitin + doxycycline	③④
HHF (2017) [123]	60/60	21	FKQJP + C	Levofloxacin	①②③④
WJF (2019) [124]	60/60	21	FKQJP + C	Levofloxacin	①③④
MTX (2016) [125]	40/40	14/42	FKQJP + metronidazole	Metronidazole + medroxyprogesterone	①③⑤
SXW (2018) [126]	45/45	42	FKQJP + metronidazole	Metronidazole + medroxyprogesterone	①②④
ZHH (2018) [127]	52/52	42	FKQJP + metronidazole	Metronidazole + medroxyprogesterone	①③⑤
ZL (2019) [128]	34/34	30	FKQJP + metronidazole	Metronidazole + medroxyprogesterone	①③④
DCL (2017) [129]	20/20	42	FKQJP + metronidazole	Metronidazole + medroxyprogesterone	①
DFY (2017) [130]	80/80	42	FKQJP + metronidazole	Metronidazole + medroxyprogesterone	①③⑤
GY (2015) [131]	78/78	14/42	FKQJP + metronidazole	Metronidazole + medroxyprogesterone	①④
LWP (2019) [132]	39/39	30	FKQJP + metronidazole	Metronidazole + medroxyprogesterone	①
MYJ (2018) [133]	42/42	42	FKQJP + metronidazole	Metronidazole + medroxyprogesterone	①
DFL (2017) [134]	41/41	42	FKQJP + metronidazole	Metronidazole + medroxyprogesterone	①③④
HXY (2018) [135]	50/50	14/42	FKQJP + metronidazole	Metronidazole + medroxyprogesterone	①④
ZQ (2019) [136]	40/40	42	FKQJP + metronidazole	Metronidazole + norethisterone	①③④
LXZ (2017) [137]	64/64	14	FKQJP + C	Metronidazole + medroxyprogesterone	①②③
CXH (2017) [138]	30/30	60	FKQJP + C	Metronidazole + medroxyprogesterone	①
FCF (2016) [139]	90/90	14/42	FKQJP + C	Metronidazole + medroxyprogesterone	①③④
LBY (2019) [140]	30/30	14/42	FKQJP + C	Metronidazole + medroxyprogesterone	①③④
LSQ (2017) [141]	45/44	42	FKQJP + C	Metronidazole + medroxyprogesterone	①②③④
YL (2018) [142]	40/40	14/42	FKQJP + C	Metronidazole + medroxyprogesterone	①④
ZQ (2016) [143]	156/156	14/60	FKQJP + C	Metronidazole + medroxyprogesterone	①④⑤
ZCY (2018) [144]	14/14	42	FKQJP + C	Metronidazole + medroxyprogesterone	①④
ZGB (2017) [145]	41/41	42	FKQJP + C	Metronidazole + medroxyprogesterone	①
GLJNT (2017) [146]	90/90	42	FKQJP + C	Metronidazole + norethisterone	①③
CJH (2016) [147]	40/40	42	FKQJP + C	Metronidazole + norethisterone	①③
AYGL (2018) [148]	40/40	42	FKQJP + C	Metronidazole + norethisterone	③④
SXX (2018) [149]	70/70	42	FKQJP + C	Metronidazole + norethisterone + vaginal douche	①③④
XCY (2015) [150]	75/75	21	FKQJP + C	Metronidazole + norethisterone + vaginal douche	①③④
HF (2016) [151]	75/75	21	FKQJP + C	Metronidazole + norethisterone + vaginal douche	①④

*Note.* ①, total effective rate; ②, incidence of adverse reaction; ③, the time of clinical symptom remission/disappearance; ④, serum inflammatory factor concentration. FKQJP, Fuke Qianjin tablet; C, control group regimens.

**Table 3:** Risk of bias evaluation of included literature.

Study ID	Random sequence generation (selection bias)	Allocation concealment (selection bias)	Blinding of participants and personnel (performance bias)	Blinding of outcome assessment (detection bias)	Incomplete outcome data (attrition bias)	Selective reporting (reporting bias)	Other bias
*Acute pelvic inflammatory*							
CCJ (2018) [27]	U	U	U	U	L	L	U
YML (2013) [28]	U	U	U	U	L	L	U
ZJ (2017) [29]	L	U	U	U	L	L	U
DY (2014) [30]	U	U	U	U	L	L	U
SL (2015) [31]	U	U	U	U	L	L	U
TSQ (2019) [32]	U	U	U	U	L	L	U
XQX (2014) [33]	U	U	U	U	L	H	U
YFY (2013) [34]	U	U	U	U	L	L	U
RS (2012) [35]	U	U	U	U	L	L	U
SFM (2015) [36]	U	U	U	U	L	L	U
ZY (2013) [37]	U	U	U	U	L	L	U
FXH (2018) [38]	L	U	U	U	L	L	U
SYQ (2015) [39]	U	U	U	U	L	L	U
ZHY (2012) [40]	U	U	U	U	L	L	U
ZYZ (2012) [41]	U	U	U	U	L	L	U
YCQ (2017) [42]	U	U	U	U	L	L	U
LWR (2013) [43]	U	U	U	U	L	L	U
WGJ (2011) [44]	U	U	U	U	L	L	U
HRM (2013) [45]	U	U	U	U	L	L	U
GSY (2012) [46]	U	U	U	U	L	L	U
ZYH (2016) [47]	U	U	U	U	L	L	U
ZNN (2012) [48]	U	U	U	U	L	L	U
LJ (2014) [49]	U	U	U	U	L	L	U

*Chronic pelvic inflammatory*							
LJX2 (2014) [50]	U	U	U	U	L	L	U
XZJ (2007) [51]	H	U	U	U	L	L	U
CXC (2019) [52]	U	U	U	U	L	L	U
DJL (2014) [53]	U	U	U	U	L	L	U
PGZ (2016) [54]	H	U	U	U	L	L	U
SXL (2015) [55]	L	U	U	U	L	L	U
WQF (2013) [56]	U	U	U	U	L	L	U
ZXJ (2015) [57]	L	U	U	U	L	L	U
ZMY (2017) [58]	U	U	U	U	L	L	U
LJX1 (2014) [50]	U	U	U	U	L	L	U
LXX (2019) [59]	L	U	U	U	L	L	U
LH (2019) [60]	U	U	U	U	L	L	U
PJQ (2019) [61]	U	U	U	U	L	L	U
SWA (2014) [62]	U	U	U	U	L	L	U
SQB2 (2012) [63]	U	U	U	U	L	L	U
ZY (2014) [64]	U	U	U	U	L	L	U
SQB1 (2012) [63]	U	U	U	U	L	L	U
SPF (2015) [65]	U	U	U	U	L	L	U
CF (2018) [66]	U	U	U	U	L	L	U
DLL (2017) [67]	U	U	U	U	L	L	U
FJY (2010) [68]	U	U	U	U	L	L	U
FP (2013) [69]	U	U	U	U	L	L	U
KY (2018) [70]	U	U	U	U	L	L	U
LMY (2016) [71]	U	U	U	U	L	L	U
LYK (2016) [72]	U	U	U	U	L	L	U
LongH (2011) [73]	U	U	U	U	L	L	U
RDQ (2019) [74]	U	U	U	U	L	L	U
ShaoXL (2012) [75]	U	U	U	U	L	L	U
TXG (2015) [76]	U	U	U	U	L	L	U
WJP (2013) [77]	U	U	U	U	H	L	U
YM (2017) [78]	U	U	U	U	L	L	U
YQ (2014) [79]	U	U	U	U	L	L	U
ZYL (2017) [80]	L	U	U	U	L	L	U
DLH (2019) [81]	H	U	U	U	L	L	U
HJY (2019) [82]	L	U	U	U	L	L	U
LJ (2019) [83]	U	L	L	U	L	L	U
SJL (2016) [84]	U	U	U	U	L	L	U
WJ (2018) [85]	L	U	U	U	L	L	U
ZL (2015) [86]	U	U	U	U	L	L	U
ZT (2018) [87]	U	U	U	U	L	L	U
ZhaoJ (2017) [88]	U	U	U	U	L	L	U
ZengJ (2016) [89]	U	U	U	U	L	L	U
CXF (2018) [90]	U	U	U	U	L	L	U
DCM (2016) [91]	U	U	U	U	L	L	U
DQY (2015) [92]	U	U	U	U	L	L	U
HSX (2019) [93]	H	U	U	U	L	L	U
HHJ (2019) [94]	L	U	U	U	L	L	U
HHM (2019) [95]	L	U	U	U	L	L	U
HYL (2015) [96]	L	U	U	U	L	L	U
LML (2019) [97]	U	U	U	U	L	L	U
LHR (2013) [98]	U	U	U	U	L	L	U
LXL (2018) [99]	L	U	U	U	L	L	U
LSY (2018) [100]	L	U	U	U	L	L	U
LYZ (2012) [101]	H	U	U	U	L	L	U
MYQ (2019) [102]	U	U	U	U	L	L	U
RC (2016) [103]	U	U	U	U	L	L	U
RL (2017) [104]	L	U	U	U	L	L	U
SNY·HSY (2016) [105]	U	U	U	U	L	L	U
SQJ (2012) [106]	U	U	U	U	L	L	U
YLH (2012) [107]	U	U	U	U	L	L	U
WDD (2017) [108]	L	U	U	U	L	L	U
WHL (2011) [109]	U	U	U	U	L	L	U
WLY (2019) [110]	U	U	U	U	L	L	U
WSY (2011) [111]	U	U	U	U	L	L	U
WY (2018) [112]	L	U	U	U	L	L	U
WDM (2019) [113]	U	U	U	U	L	L	U
XW (2015) [114]	U	U	U	U	L	L	U
XRQ (2018) [115]	U	U	U	U	L	L	U
YDM (2015) [116]	U	U	U	U	L	L	U
YXH (2016) [117]	L	U	U	U	L	L	U
ZhangJ (2017) [118]	U	U	U	U	L	L	U

*Endometritis*							
SFF (2017) [119]	U	U	U	U	L	L	U
SCP (2019) [120]	U	U	U	U	L	L	U
DYY (2019) [121]	U	U	U	U	L	L	U
LY (2019) [122]	U	U	U	U	L	L	U
HHF (2017) [123]	L	U	U	U	L	L	U
WJF (2019) [124]	U	U	U	U	L	L	U
MTX (2016) [125]	U	U	U	U	L	L	U
SXW (2018) [126]	L	U	U	U	L	L	U
ZHH (2018) [127]	L	U	U	U	L	L	U
ZL (2019) [128]	L	U	U	U	L	L	U
DCL (2017) [129]	U	U	U	U	L	L	U
DFY (2017) [130]	L	U	U	U	L	L	U
GY(2015) [131]	L	U	U	U	L	L	U
LWP (2019) [132]	L	U	U	U	L	L	U
MYJ (2018) [133]	L	U	U	U	L	L	U
DFL (2017) [134]	U	U	U	U	L	L	U
HXY (2018) [135]	U	U	U	U	L	L	U
ZQ (2019) [136]	U	U	U	U	L	L	U
LXZ (2017) [137]	U	U	U	U	L	L	U
CXH (2017) [138]	L	U	U	U	L	L	U
FCF (2016) [139]	U	U	U	U	L	L	U
LBY (2019) [140]	U	U	U	U	L	L	U
LSQ (2017) [141]	L	U	U	U	L	L	U
YL (2018) [142]	U	U	U	U	L	L	U
ZQ (2016) [143]	U	U	U	U	L	L	U
ZCY (2018) [144]	U	U	U	U	L	L	U
ZGB (2017) [145]	U	U	U	U	L	L	U
GLJNT (2017) [146]	U	U	U	U	L	L	U
CJH (2016) [147]	U	U	U	U	L	L	U
AYGL (2018) [148]	L	U	U	U	L	L	U
SXX (2018) [149]	U	U	U	U	L	L	U
XCY (2015) [150]	L	U	U	U	L	L	U
HF (2016) [151]	L	U	U	U	L	L	U

L, low risk; U, unclear risk; H, high risk.

**Table 4:** Meta-analysis results of primary outcomes.

Indication	Type of intervention		Number of studies included	Cases	Heterogeneity test		Effect model	Results of the meta-analysis		
	Experimental group	Control group			*I* ^*2*^	*p*		OR (95% CI)	*z*	*p*
Acute PID	Total effective rate		23		0.0%	0.999	F	5.57 (4.09, 7.58)	10.90	0.001
	FKQJP + control group	Penicillin	6	549	0.0%	0.711	F	5.77 (3.17, 10.49)	5.74	0.001
	FKQJP + control group	Penicillin + metronidazole	7	801	0.0%	0.887	F	7.80 (4.00, 15.18)	6.04	0.001
	FKQJP + control group	Quinolones	2	206	0.0%	0.584	F	3.99 (1.81, 8.77)	3.44	0.001
	FKQJP + control group	Quinolones + nitroimidazole	2	140	0.0%	0.773	F	7.23 (1.78, 29.28)	2.77	0.006
	FKQJP + control group	Roxithromycin	2	180	0.0%	0.809	F	4.59 (1.63, 12.96)	2.88	0.004
	FKQJP + control group	Metronidazole	2	132	0.0%	0.330	F	2.96 (1.07, 8.22)	2.08	0.037
	FKQJP + moxifloxacin	Ceftriaxone + tinidazole	2	220	0.0%	0.829	F	5.90 (2.45, 14.20)	3.96	0.001
	Incidence of adverse reaction	10	10	958	0.0%	0.989	F	0.71 (0.45, 1.10)	1.55	0.121
Chronic PID	Total effective rate		69	8478	0.0%	0.999	F	4.70 (4.07, 5.42)	21.21	0.001
	FKQJP	*β*-Lactam + nitroimidazole	2	284	0.0%	0.735	F	2.55 (1.52, 4.28)	3.53	0.001
	FKQJP + control group	*β*-Lactam	7	626	0.0%	0.939	F	3.88 (2.34, 6.45)	5.24	0.001
	FKQJP + control group	*β*-Lactam + nitroimidazole	7	700	0.0%	0.918	F	6.27 (3.46, 11.37)	6.04	0.001
	FKQJP+*β*-lactam + nitroimidazole	FKQJP	2	250	0.0%	0.579	F	7.80 (3.29, 18.50)	4.66	0.001
	FKQJP + control group	Macrolide	15	1905	0.0%	0.869	F	3.54 (2.67, 4.68)	8.81	0.001
	FKQJP + control group	Quinolone	8	892	0.0%	0.925	F	5.54 (3.61, 8.52)	7.82	0.001
	FKQJP + control group	Quinolone + nitroimidazole	30	3821	0.0%	1.000	F	5.67 (4.49, 7.16)	14.56	0.001
	Incidence of adverse reaction		30	3082	0.0%	0.708	F	0.60 (0.46, 0.80)	3.55	0.001
Endometritis	Total effective rate		30	3521	0.0%	0.998	F	5.09 (4.03, 6.43)	13.63	0.001
	FKQJP + control group	Antibiotics	4	680	0.0%	0.979	F	9.01 (4.23, 19.21)	5.69	0.001
	FKQJP + antibiotics	Antibiotics + progesterone	12	1122	0.0%	0.969	F	4.84 (3.25, 7.21)	7.77	0.001
	FKQJP + control group	Antibiotics + progesterone	11	1279	0.0%	0.959	F	4.40 (3.03, 6.39)	7.78	0.001
	FKQJP + control group	Antibiotics + progesterone + vaginal douche	3	440	0.0%	0.758	F	5.25 (2.92, 9.44)	5.54	0.001

FKQJP, Fuke Qianjin tablet; R, random-effect model; F, fix-effect model.

**Table 5:** The meta-analysis results of the secondary outcomes.

Indication	Outcomes	Number of studies included	Cases	Heterogeneity test		Effect model	Results of the meta-analysis	
				*I* ^*2*^	*p*		SMD/OR (95% CI)	*p*
Acute PID	Clinical symptom remission or disappearance time	8	899	—	—	—	—	—
	The time of the masses disappearance	7	821	89.2%	0.001	R	−1.36 (−1.84, −0.89)	0.001
	The time of body temperature return to normal	7	821	84.9%	0.001	R	−1.19 (−1.58, −0.79)	0.001
	The time to relieve abdominal pain	7	821	90.7%	0.001	R	−1.83 (−2.38, −1.29)	0.001
	Serum inflammatory factor concentration	6	558	—	—	—	—	—
	CRP concentration	3	300	92.1%	0.001	R	−1.99 (−3.01, −0.98)	0.001
	TNF-*α* concentration	4	372	50.8%	0.107	F	−1.73 (−1.97, −1.49)	0.001
	IL-6 concentration	4	380	94.4%	0.001	R	−1.53 (−2.52, −0.53)	0.001
Chronic PID	Clinical symptom remission or disappearance time	13	1394	—	—	—	—	—
	The remission time of uterine pain	5	542	92.0%	0.001	R	−2.48 (−3.29, −1.66)	0.001
	The remission time of abdominal pain	12	1208	85.6%	0.001	R	−2.12 (−2.49, −1.74)	0.001
	The time for leucorrhoea to return to normal	7	782	27.4%	0.219	F	−1.38 (−1.54, −1.22)	0.001
	Serum inflammatory factor concentration	11	2317	—	—	—	—	—
	CRP concentration	5	1575	87.6%	0.001	R	−3.10 (−3.69, −2.51)	0.001
	TNF-*α* concentration	5	646	96.6%	0.001	R	−2.56 (−3.68, −1.43)	0.001
	IL-2 concentration	3	264	0.0%	0.969	F	0.85 (0.59, 1.10)	0.001
	IL-4 concentration	3	264	0.0%	0.933	F	0.60 (0.59, 0.85)	0.001
	IL-10 concentration	2	182	0.0%	0.790	F	0.49 (0.20, 0.79)	0.001

Endometritis	Endometrial thickness	16	1680	87.8%	0.001	R	2.25 (1.89, 2.60)	0.001
	Normalization rate of menstrual	12	1413	0.0%	0.978	F	4.01 (3.04, 5.30)	0.001
	Normalization rate of menstrual cycle	6	845	46.3%	0.097	F	5.57 (3.82, 8.13)	0.001
	Normalization rate of menstrual amount	6	865	79.9%	0.001	R	4.96 (1.72, 14.28)	0.001
	Incidence of irregular vagina bleeding	15	1693	0.0%	0.995	F	0.23 (0.16, 0.31)	0.001
	6-month relapse rate	3	576	0.0%	0.683	F	0.15 (0.07, 0.35)	0.001

FKQJP, Fuke Qianjin tablet; R, random-effect model; F, fix-effect model.

## Data Availability

The data generated or analyzed during this study are included within this published article. The data used to support the findings of this study have been deposited in the 7 electronic databases, including PubMed, The Cochrane Library, Web of science, CNKI, Wan Fang, VIP, and Sinomed.

## Abbreviations

CNKI: China National Knowledge Infrastructure
VIP: Chinese Scientific Journals Database
PID: Pelvic inflammatory disease
FKQJP: Fuke Qianjin tablet.
